# Analytical quality by design methodology for botanical raw material analysis: a case study of flavonoids in Genkwa Flos

**DOI:** 10.1038/s41598-021-91341-w

**Published:** 2021-06-07

**Authors:** Min Kyoung Kim, Sang Cheol Park, Geonha Park, Eunjung Choi, Yura Ji, Young Pyo Jang

**Affiliations:** 1 University Institute of Pharmacy, Kyung Hee University, Seoul, 02447 South Korea; 2 Department of Life and Nanopharmaceutical Sciences, Graduate School, Kyung Hee University, Seoul, 02447 South Korea; 3 Department of Oriental Pharmaceutical Science, College of Pharmacy, Kyung Hee University, Seoul, 02447 South Korea

**Keywords:** Pharmaceutics, Process chemistry

## Abstract

The present study introduces a systematic approach using analytical quality by design (AQbD) methodology for the development of a qualified liquid chromatographic analytical method, which is a challenge in herbal medicinal products due to the intrinsic complex components of botanical sources. The ultra-high-performance liquid chromatography-photodiode array-mass spectrometry (UHPLC-PDA-MS) technique for 11 flavonoids in Genkwa Flos was utilized through the entire analytical processes, from the risk assessment study to the factor screening test, and finally in method optimization employing central composite design (CCD). In this approach, column temperature and mobile solvent slope were found to be critical method parameters (CMPs) and each of the eleven flavonoid peaks’ resolution values were used as critical method attributes (CMAs) through data mining conversion formulas. An optimum chromatographic method in the design space was calculated by mathematical and response surface methodology (RSM). The established chromatographic condition is as follows: acetonitrile and 0.1% formic acid gradient elution (0–13 min, 10–45%; 13–13.5 min, 45–100%; 13.5–14 min, 100–10%; 14–15 min, 10% acetonitrile), column temperature 28℃, detection wavelength 335 nm, and flow rate 0.35 mL/min using C_18_ (50 × 2.1 mm, 1.7 μm) column. A validation study was also performed successfully for apigenin 7-*O*-glucuronide, apigenin, and genkwanin. A few important validation results were as follows: linearity over 0.999 coefficient of correlation, detection limit of 2.87–22.41, quantitation limit of 8.70–67.92, relative standard deviation of precision less than 0.22%, and accuracy between 100.13 and 102.49% for apigenin, genkwanin, and apigenin 7-*O*-glucuronide. In conclusion, the present design-based approach provide a systematic platform that can be effectively applied to ensure pharmaceutically qualified analytical data from complex natural products based botanical drug.

## Introduction

Interest in high-level analytical system for complex pharmaceutical ingredients such as plant extract is increasing in the reality that drug development using natural extracts is increasing worldwide. Botanical drug guidelines of the United States Food and Drug Administration (USFDA) which was revised in 2016, recommends a ‘Totality-of-the-Evidence’ approach that comprehensively utilizes fingerprint analysis, chemical identification, and quantification of active or chemical constituents in the drug substance to characterize the complexity of the botanical sources to ensure consistency in drug quality^1,2^.

In order to achieve high standard of analytical methods of quality control, quality by design (QbD) approach have been adopted during analytical method development of various pharmaceutical practices^3–6^. The QbD is a disciplined approach to understand and control new drug products, based on sound science and quality risk management in diverse pharmaceutical processes^7,8^. Analytical methods play a significant role in drug product development in the control scheme of constant quality system monitoring of a product lifecycle^9^. The International Conference on Harmonization (ICH) is preparing to develop a new ICH Quality Guideline (ICH Q14) on Analytical Procedure Development, which will include the QbD concept for analytical methods, termed Analytical Quality by Design (AQbD)^10^. The AQbD approach begins with determining the analytical target profile (ATP), which is the prospective target of the analytical method development process and relates performance elements based on the intended target criteria^11^. The selection of critical method attributes (CMAs) is also performed, which directly represent a strong link to the intended criteria such as selectivity, precision, or accuracy in the desired analytical quality. Secondly, parameters that may affect analytical results are identified through a risk assessment approach^10^. Those highly selected risk factors are known as critical method parameters (CMPs) which should be tested with design of experiment (DoE) methodology and statistical screening. Thirdly, the polynomial relationships between CMAs and CMPs were studied in order to understand the in-depth cause-effect aspects that were statistically designed to identify the influential input variables affecting the representative output variables^12^. Meanwhile, the DoE is usually conducted twice by screening factors and then response surface methodology for optimizing the analytical method. The purpose of the screening study is to find the high-risk factors through fewer experiments, which is usually performed with designed two-level models such as full factorial design (FFD), fractional factorial design (FrFD), and Plackett-Burman design (PBD)^8,12^. In addition, an optimization process is conducted to ensure that proper quality is attained in the analytical method by considering the selected high-risk factors during design. The results are interpreted through response surface methodology (RSM) which is a potent statistical technique in mathematical modeling to interpret the designed-responses. Optimized strategic design responses include Box–Behnken design (BBD), central composite design (CCD), Taguchi design (TD), Mixture design, and Doehlert design^8,12^. Finally, the most appropriate designed point or method operable design region (MODR) is calculated from the RSM and confirmed by the method validation processes^13^.

While quality control systems based on the AQbD approach are applied widely in the field of pharmaceuticals, few application studies have been conducted on botanical extracts^14–16^. Since botanical extracts have complex and diverse phytochemicals as active ingredients, the selection of optimal analytical conditions is not simple. Also, it is quite challenging to screen the analytical parameters (i.e. buffer pH, organic solvent type, gradient slope, column temperature, etc.) that must be optimized by DoE technique.

In this paper, a systematic design-based approach to optimize a liquid chromatographic analytical method for major constituents of Genkwa Flos was investigated to suggest an analytical platform for how to consider CMAs and identify CMPs in an integrated case study with a botanical source. Compared to the routine One-Factor-At-a-Time (OFAT) approach, which tried one variable and collected one response, the current total quality approach utilizes scientific designing models and statistic expertise to finally obtain less experiment time, robust, precise, and easily validated analytical method^9^.

The flower buds of *Daphne genkwa* (Genkwa Flos, Thymelaeaceae) have been widely used as traditional oriental medicine in East Asia, China and Korea, and continue to draw great attention for their diverse pharmacological efficacy^17–19^. Previous phytochemical studies on *D. genkwa* revealed diverse chemical components including diterpenoids, flavonoids, lignans, and coumarins^20–22^. In recent years, genkwa flavonoids, as the main active constituents of Genkwa Flos, have been reported to exhibit remarkable pharmacological activities such as anti-inflammatory^23^, immunoregulation^24^, anti-tumor activity in colorectal cancer^25^, and anti-rheumatoid arthritis activity^26^. In order to exploit Genkwa Flos as a main ingredient of botanical drug, it is necessary to develop a robust and reliable analytical method for quality control, which is able to identify and quantify multiple components in botanical extracts in order to assure the consistency of pharmacological efficacy of herbal drug products.

CMPs were determined by risk assessment and factor screening experimental data in sequence. CMAs were established by equations that can be expressed as a single number by collecting the resolution of multiple peaks. After developing the optimized method by central composition design (CCD), the method validation was carried out in order to evaluate the soundness of the methods.

## Results and discussion

### Characterization of flavonoids using UHPLC-PDA-MS analysis

**Figure 1:**
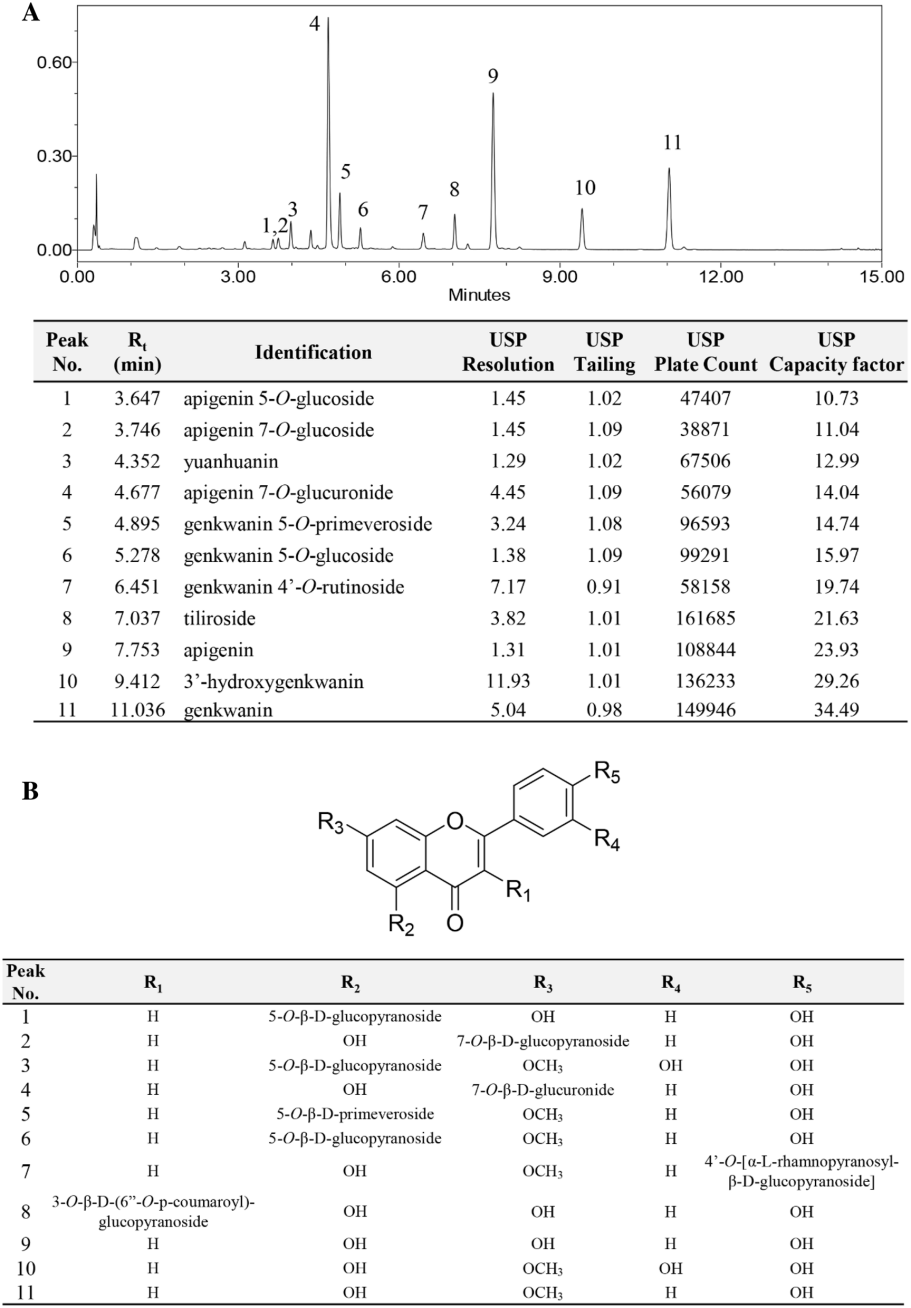
Representative UHPLC chromatogram of Genkwa Flos extract tagged with characteristic 11 flavonoid peaks (**A**) and their chemical structures (**B**). Kinetex-C_18_ 50× 2.1 mm, 1.7 μm column; mobile phase-A: 0.1% formic acid in water, mobile phase-B: acetonitrile; 335 nm detection; column temperature 28 ℃; 0.35 mL/min; gradient Time (min):%B, 0:10, 13:45, 13.5:100, 14:10, 15:10 used for the chromatogram.

UHPLC-PDA-MS system was utilized for the identification of flavonoids in Genkwa Flos. High-resolution mass data from Time-of-Flight (TOF) analyzer combined with UV–Visible absorption spectral pattern enabled to identify known flavonoids from Genkwa Flos extracts by direct comparison with those of previous researches^22,23^ and/or reference standard solutions. A total of eleven identified flavonoids were listed in Table 1 providing their retention time, λ_max_, quasi-molecular ion, observed mass, mass difference, and molecular formula. Those were also tagged as peak 1 to peak 11 in the UHPLC chromatogram obtained at 335 nm (Fig. 1A) which are apigenin 5-*O*-glucoside, apigenin 7-*O*-glucoside, yuanhuanin, apigenin 7-*O*-glucuronide, genkwanin 5-*O*-primeveroside, genkwanin 5-*O*-glucoside, genkwanin 4′-*O*-rutinoside, tiliroside, apigenin, 3′-hydroxygenkwanin, and genkwanin as eluted in order.

**Table 1:** The retention time, λ_max,_ quasi-molecular ion, observed mass, mass difference, and molecular formulae of eleven peaks by UPLC-PDA-ESI/MS analysis.

Peak no.	R_t_ (min)	λ_max_	Quasi-molecular ion	Observed mass (*m/z*)	Mass difference (mmu)	Molecular formula	Identification	References
1	3.647	260.6	[M + H]^+^	433.1143	0.9	C_21_H_20_O_10_	Apigenin 5-*O*-glucoside	Du et al.^23^
		335.3						
2	3.746	255.1	[M + H]^+^	433.1141	0.7	C_21_H_20_O_10_	Apigenin 7-*O*-glucoside	Du et al.^23^
		348.4						
3	4.352	241.0	[M + H]^+^	463.1239	0.1	C_22_H_22_O_11_	Yuanhuanin	Wang et al.^22^
		340.9						
4	4.677	266.2	[M + H]^+^	447.0926	0.1	C_21_H_18_O_11_	Apigenin 7-*O*-glucuronide	*Ref. std.
		337.7						
5	4.895	261.3	[M + H]^+^	579.1755	4.1	C_27_H_30_O_14_	Genkwanin 5-*O*-primeveroside	Wang et al.^22^
		332.8						
6	5.278	261.3	[M + H]^+^	447.1275	− 1.6	C_22_H_22_O_10_	Genkwanin 5-*O*-glucoside	Du et al.^23^
		332.2						
7	6.451	253.3	[M + H]^+^	593.1877	0.7	C_28_H_32_O_14_	Genkwanin 4′-*O*-rutinoside	Wang et al.^22^
		348.4						
8	7.037	266.2	[M + H]^+^	595.1426	− 2.5	C_30_H_26_O_13_	Tiliroside	Du et al.^23^
		314.8						
9	7.753	266.8	[M + H]^+^	271.0620	1.4	C_15_H_10_O_5_	Apigenin	*Ref. std.
		338.4						
10	9.412	253.3	[M + H]^+^	301.0715	0.3	C_16_H_12_O_6_	3′-Hydroxygenkwanin	Du et al.^23^
		348.4						
11	11.036	267.4	[M + H]^+^	285.0752	− 1.1	C_16_H_12_O_5_	Genkwanin	*Ref. std.
		337.7						

*Ref.std.; comparison with those reference standard solutions by UHPLC analysis.

*The peak number and retention time information are tagged on representative chromatogram in Fig. 1.

### Analytical target profile (ATP) and critical method attributes (CMAs)

The first step in AQbD-based method development is to define the ATP for stepwise and scientific procedures^7^. An analytical procedure which is able to quantitatively determine the specified eleven flavonoids in Genkwa Flos is a target of this study. Various elements of ATP such as analytical technique and instrument requirement were summarized as the intended target criteria (Supplementary Table S1). After ATP set-up, the potential CMAs were considered based on preliminary studies and review of the literature^8,9^. The general key CMA is the resolution (*R*_*s*_) of critical peaks^4,15,27^, which may be a critical attribute to avoid peak overlap for selective identification in liquid chromatography. Finally, the CMAs, corresponding to ATP, were established as countable peak number (Y_n_) and resolution (Y_1–11_ and Y_sum_) after substantial consideration based on the modeling of experimental studies.

### Preliminary studies

To carry out design-based method development studies, several preliminary tests were performed in different columns (i.e., length, particle size, manufacturer), using various solvents (i.e., acetonitrile, methanol), and acidified water (i.e., non-acidified, 0.1% acetic acid, 0.1% formic acid). Also, the detection wavelength for analyte was tested to acquire the greatest specific detection. The purpose of these attempts is to reduce variables by fixing those three parameters, but guarantee the best peak symmetry with the least working time. The achievement results were organized in Supplementary Table S2, and final decision to C_18_ (50 × 2.1 mm, 1.7 μm) column, acetonitrile and 0.1% formic acid water solvent system, and 335 nm detection wavelength, respectively.

### Risk assessment studies

**Figure 2:**
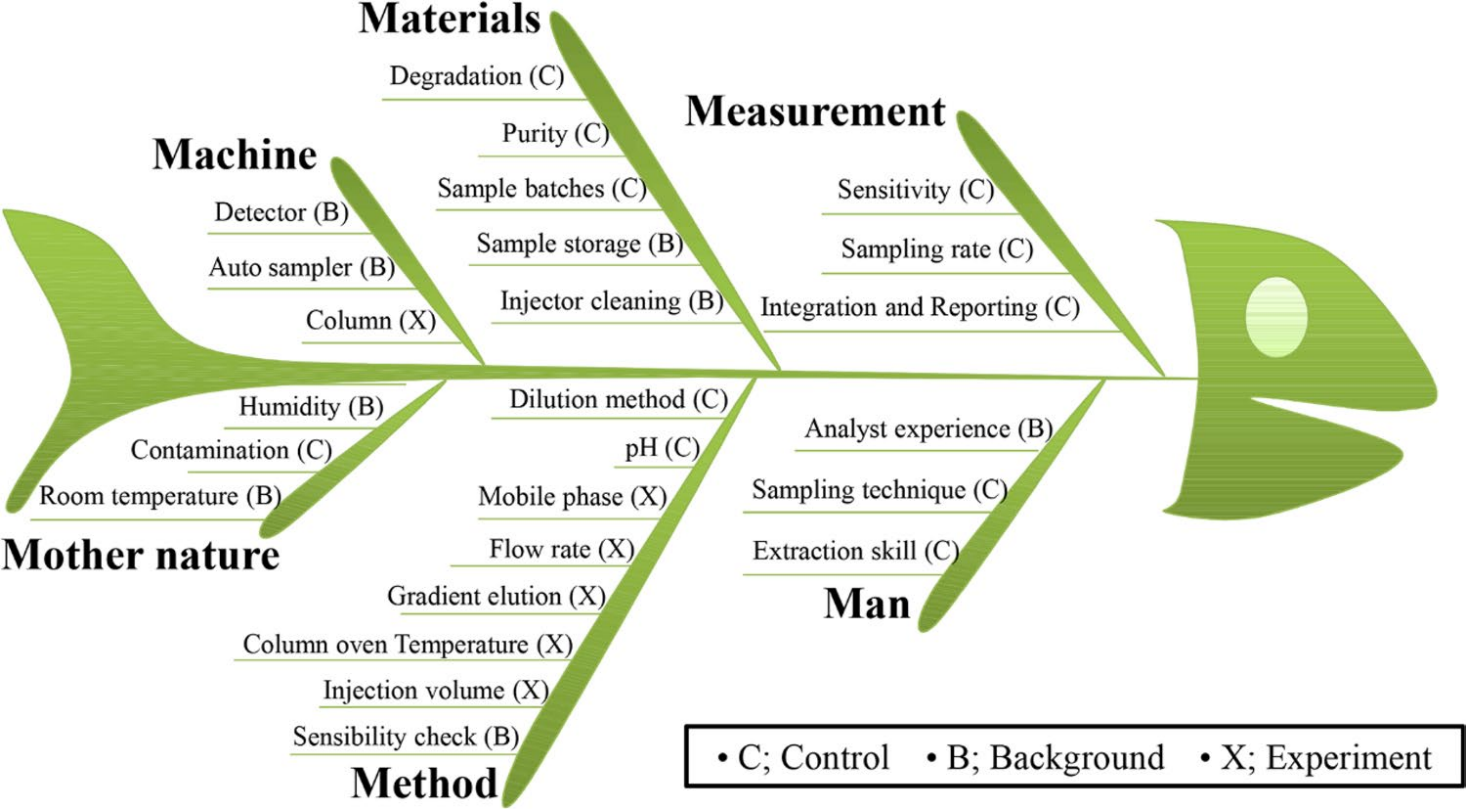
Ishikawa Fishbone in Six Sigma of the UHPLC-PDA performance.

Quality risk management (QRM) allows us to control the entire process and recognize high-risk parameters that will affect the final quality of the analytical method^28^. We endeavored to establish QRM through risk assessment studies including experimental instruments and analytical parameters as shown in Fig. 2, an Ishikawa fishbone cause-effect diagram. From the cause-effect diagram, potential factors in performing liquid chromatography could be identified and a subsequent step, the organized failure effect in each of the potential factors were calculated with a risk priority number (RPN) to sort out the high risk factors^29^. Following the guidance of ICH Q11^30^, RPN numbers were calculated with the equation ‘Severity $\times$ Probability $\times$ Detectability’ to allocate risk in each failure mode. The risk assessment and control strategy are summarized in Table 2. Those parameters, column temperature (X_1_), flow rate (X_2_), injection volume (X_3_), and gradient slope, indicate highly influential factors, which are calculated greater than 10 RPN. Practically, when designing the models, the gradient slope was converted into run time (X_4_), because the initial and final percentages of acetonitrile solvent were fixed at 10 to 45 (Table 3). Thus, these four parameters were thereby selected for the further factor screening studies. The parameters counted less than 10 RPN were controlled as the constant.

**Table 2:** Risk assessment and control strategy for AQbD-enabled development UHPLC-PDA method for Genkwa Flos.

Potential failure cause	Failure effect	Risk mitigation	P	S	D	RPN
Injection volume*	Change the peak resolutions and S/N	Optimized by DoE and control	3	2	3	18
Sample stability	Change in peak resolutions and S/N	Ascertain the stability of prepared sample solutions	1	1	2	2
Mobile phase	Change in peak symmetry and chromatography	At least four mobile phases were tested	2	2	2	8
Columns	Lot variability may change	At least three columns were tested	2	2	2	8
Vials	Exposure to light results in an increase of impurity	Amber vials to be used	1	2	1	2
Humidity	Change in weighing	Standard operating procedures to be followed to dry the samples	1	2	2	4
Column temperature*	Changes in peak resolutions, elute time, and S/N	Optimized by DoE and control	3	2	2	12
Sample temperature	May change the peak resolutions	Control autosampler temperature at 20℃	2	1	2	4
Misidentification of peaks	Incorrect values reported	Training, example chromatograph	3	2	1	6
Gradient slope*	Changes in whole chromatography	Optimized by DoE and control	4	2	3	24
Flow rate*	Changes in peak resolutions and elute time	Optimized by DoE and control	2	2	3	12
Instrument model	Changes in whole chromatography	UHPLC system was selected	2	2	2	8

*S/N* signal to noise, *DoE* design of experiments, *P* probability, *S* severity, *D* detectability.

Risk priority number (RPN) = Severity $\times$ Probability $\times$ Detectability.

*High risk factors selected by upper 10 RPN.

**Table 3:** 4^2^-Full factorial design (FFD) matrix for factor screening and the studied responses.

Runs	X_1_	X_2_	X_3_	X_4_	Y_n_
1	45	0.3	1.5	12	23
2	25	0.3	0.5	12	24
3	35	0.35	1	8	23
4	25	0.4	0.5	4	23
5	45	0.3	1.5	4	21
6	25	0.4	0.5	12	23
7	35	0.35	1	8	23
8	45	0.3	0.5	4	21
9	25	0.3	0.5	4	23
10	25	0.4	1.5	12	22
11	25	0.4	1.5	4	23
12	45	0.4	1.5	4	22
13	25	0.3	1.5	4	23
14	25	0.3	1.5	12	24
15	35	0.35	1	8	23
16	45	0.4	0.5	4	21
17	45	0.4	0.5	12	23
18	45	0.3	0.5	12	24
19	45	0.4	1.5	12	23

Levels of the factors studied		Range levels			
Factors	Code	Low (− 1)	Central (0)	High (+ 1)	
Column temperature (℃)	X_1_	25	35	45	
Flow rate (mL/min)	X_2_	0.30	0.35	0.40	
Injection volume (μL)	X_3_	0.5	1.0	1.5	
Run time (min)	X_4_	4	8	12	

Time (min)	% Acetonitrile	% Water (0.1% formic acid)			
**Gradient system for X**_**4**_					
0	10	90			
X_4_	45	55			
X_4_ + 0.5	100	0			
X_4_ + 1.0	10	90			
X_4_ + 2.0	10	90			

*Y*_*n*_ peak numbers.

### Factor screening studies

A (4^2^) full factorial design (FFD), 4-factors and 2-levels, was performed for finding relatively fewer significant parameters from a list of higher risk potentially affecting the chosen CMAs, peak numbers (Y_n_). Since Y_n_ generally reflects the integral quality of chromatographic separation, we chosen it for the FFD which is roughly executed at just 2-levels (Low and High). The selected high risk factors during risk assessment studies were identified as column temperature (X_1_), flow rate (X_2_), injection volume (X_3_), and run time (X_4_). The main effect(s) were estimated by selecting the first-order polynomial models, which were drawn out per Eq. (1):

1 $$ Y_{n} = 14.58 - 0.0438X_{1} - 3.75X_{2} - 0.125X_{3} + 0.1406X_{4}. $$

**Figure 3:**
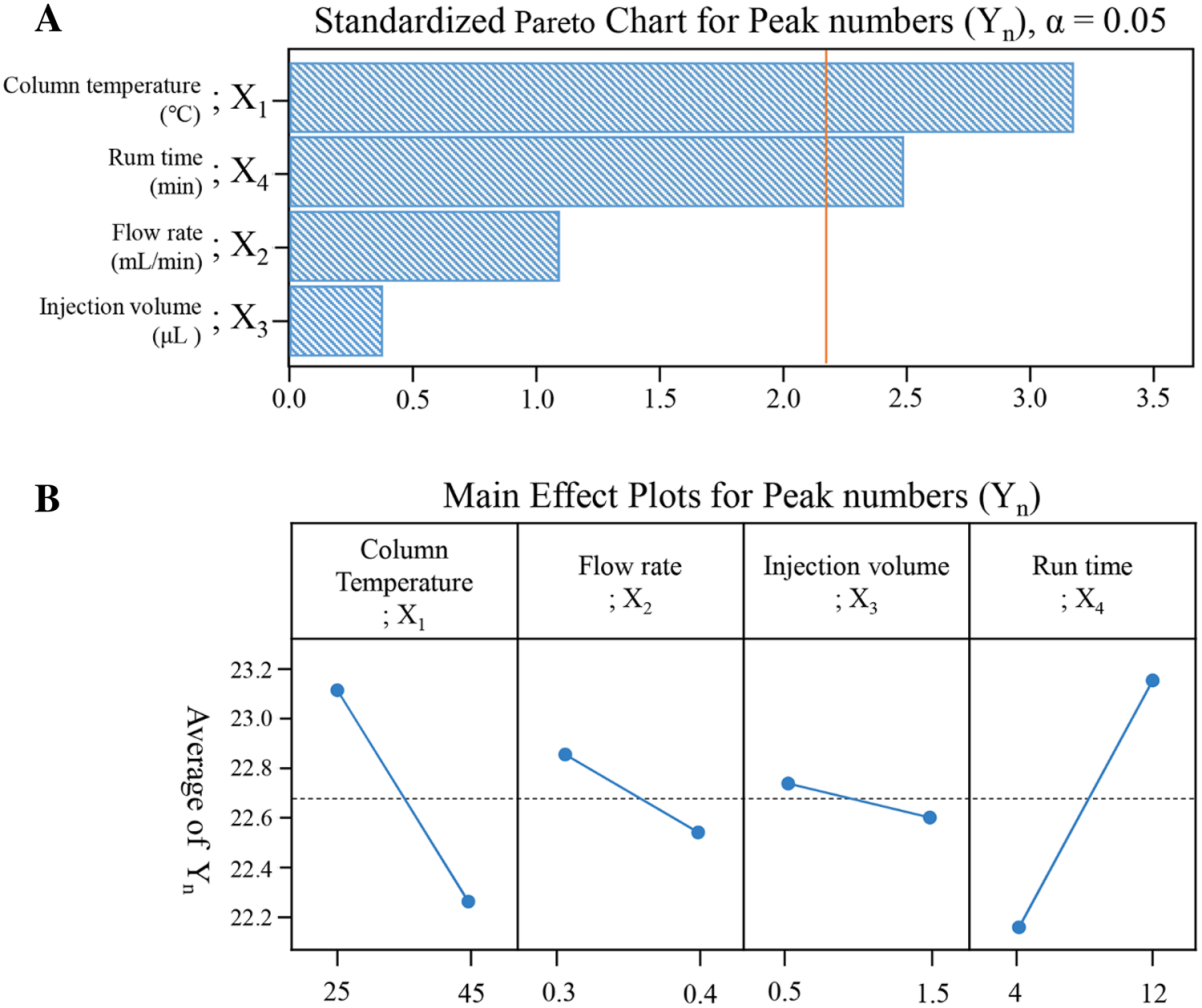
Pareto chart (**A**) and main effect plots (**B**) obtained during factor screening of critical method attributes (CMAs), *Y*_*n*_ peak numbers.

In the equation, Y_n_ is the studied CMAs, which is number of countable flavonoid peaks, when examined in each of 19 runs as depicted in Table 3. Those experimental runs were constructed randomly. A Pareto chart and Main effect plots (Fig. 3) show the significant influence of column temperature (X_1_) and run time (X_4_) on the studied CMAs, as these parameter frequencies were found to cross the corresponding *α*-value. As observed in Fig. 3B, the countable peak numbers (Y_n_) showed a negative correlation to column temperature (X_1_), but a positive effect by run time (X_4_). According to the statistical results (Table 4), the fitted model was very suitable to the experimental data by *p*-value under 0.05 with lack-of-fit larger than 0.05. Thus, factors such as column temperature (X_1_) and run time (X_4_) were selected as the CMPs for further optimization studies, and the other minor effective factors were kept as constant values. The flow rate (X_2_) was adjusted to 0.35 mL/min, while the injection volume (X_3_) was fixed at 1.0 μL.

**Table 4:** ANOVA results for response Y_n_ (peak numbers) obtained from the FFD factor screening and Y_sum_ (summarizes the eleven resolutions) obtained from the CCD response surface experimental design spaces.

Source of variations	Degree of freedom	Sum of squares	Mean squares	F-value	P-value
**ANOVA results for response Y**_**n**_** obtained from the FFD**					
Quadratic model*	4	8.7500	2.1875	4.42	0.016
Column temperature; ℃ (X_1_)*	1	3.0625	3.0625	6.18	0.026
Flow rate; mL/min (X_2_)	1	0.5625	0.5625	1.14	0.305
Injection volume; μL (X_3_)	1	0.0625	0.0625	0.13	0.728
**Run time; min (X**_**4**_)*	1	5.0625	5.0625	10.22	0.006
Lack of fit	11	6.6875	0.6080		
Total Adjusted	18	15.6842			
**ANOVA results for response Y**_**sum**_** obtained from the CCD**					
Quadratic model*	6	34.5115	5.7519	22.61	0.001
Column temperature; ℃ (X_1_)*	1	6.4929	6.4929	25.52	0.001
Run time; min (X_4_)*	1	4.4237	4.4237	17.39	0.004
X_1_∙X_1_*	1	3.0154	3.0154	11.85	0.011
X_4_∙X_4_*	1	20.3112	20.3112	79.83	0.001
X_1_∙X_4_	1	0.0606	0.0606	0.24	0.640
Lack of fit	3	1.4415	0.4805	5.66	0.064
Pure error	4	0.3395	0.0849		
Total adjusted	13	36.2926			

*Significant.

### Response surface analysis

The subsequent chromatographic method optimization was executed by selecting the second-order quadratic polynomial model, where a central composite design (CCD) model designed with level 1.41421α were conducted with fourteen experimental runs (Table 5). The analyzed CMPs were column temperature (X_1_) and run time (X_4_) and studied at five different equidistant levels, i.e. low axial (− 1.41421), low factorial (− 1), central (0), high factorial (+ 1), and high axial (+ 1.41421). Meanwhile, the potential CMAs were newly chosen as Y_1–11_, which are the resolution (*R*_s_) of each of the identified eleven flavonoid peaks listed in Table 1. Since botanical extracts have numerous phytochemicals, the resolution of each eleven peaks were defined between the closest eluted peaks. In detail, when calculate Y_8_ for the peak number 8 shown in Fig. 1A, the closest peak is just behind one eluted at 7.326 min. Besides, the first peak resolution (Y_1_) and second peak resolution (Y_2_) were of equal value, because the peaks are not totally separated or completely resolved by the UHPLC system and the closest eluting potential interference was each other. Furthermore, in several experimental runs (Table 5), the Y_1_ and Y_2_ were *R*_s_ = 0, indicating that those two peaks completely overlapped or co-eluted. The USP resolution equation using the baseline peak width drawn by lines tangent to the peak at 50% height was conducted for absolutely divided peaks, but USP Resolution (HH) using the peak width at half-height multiplied by a constant was utilized when calculated for overlapping peaks^31^.

**Table 5:** Central composite design (CCD) matrix for response surface and the studied responses.

Runs	X_1_	X_4_	Y_sum_	Each resolution (R_s_) value										
				Y_1_	Y_2_	Y_3_	Y_4_	Y_5_	Y_6_	Y_7_	Y_8_	Y_9_	Y_10_	Y_11_
1	20.86	14	18.79	1.59	1.59	1.15	1.19	2.00	2.00	1.29	2.00	2.00	2.00	1.98
2	35	14	18.18	0.85	0.85	2.00	0.85	2.00	2.00	2.00	2.00	1.64	2.00	2.00
3	35	14	18.38	0.95	0.95	2.00	0.84	2.00	2.00	2.00	2.00	1.66	2.00	2.00
4	35	14	18.87	0.99	0.99	2.00	1.04	2.00	2.00	2.00	2.00	1.83	2.00	2.00
5	49.14	14	16.49	0.00	0.00	2.00	2.00	1.80	2.00	2.00	1.98	0.71	2.00	2.00
6	35	19.66	14.14	0.00	0.00	1.06	0.62	2.00	1.31	2.00	2.00	1.15	2.00	2.00
7	35	8.34	17.94	1.45	1.45	1.06	1.54	2.00	1.79	1.84	1.29	1.50	2.00	2.00
8	35	14	18.55	0.88	0.88	2.00	0.95	2.00	2.00	2.00	2.00	1.84	2.00	2.00
9	45	18	14.06	0.00	0.00	2.00	0.93	0.93	2.00	2.00	1.11	1.09	2.00	2.00
10	45	10	15.21	0.41	0.41	2.00	0.00	1.76	2.00	2.00	1.57	1.06	2.00	2.00
11	35	14	18.36	0.94	0.94	2.00	0.84	2.00	2.00	2.00	2.00	1.64	2.00	2.00
12	25	10	16.95	0.00	0.00	2.00	2.00	1.58	1.65	2.00	2.00	1.87	2.00	1.85
13	35	14	18.38	0.95	0.95	2.00	0.84	2.00	2.00	2.00	2.00	1.64	2.00	2.00
14	25	18	16.28	1.08	1.08	1.29	1.14	1.07	2.00	2.00	1.00	1.62	2.00	2.00

**Levels of the factors studied**		Range levels												
Factors	Code	Low axial (− α, − 1.41421)	Low factorial (− 1)	Central (0)	High factorial (+ 1)	High axial (+ α, + 1.41421)								
Column temperature (℃)	X_1_	20.86	25	35	45	49.14								
Run time (min)	X_4_	8.34	10	14	18	19.66								

*Y*_*sum*_ summarizes the eleven resolutions.

In order to evaluate efficiently the total quality of separation in chromatographic fingerprints derived from each experimental run, one hypothetic score was introduced as total summation of Ys values of each peaks. In the design space, the Y_1_ to Y_11_ peaks were integrated as one value of Y_sum_ by Eq. (3), which represents the estimated response for the experimental correlation with the two selected CMPs. Also, in order to prevent the value of a few peaks from dominating the overall result, it was necessary to determine the maximum value of each variable. A resolution over 1.5 usually indicates great separation, and when it is greater than 2, the peak is considered to be completely separated^32^. Hence, before integrating, the resolution values greater than 2 were set to 2 as shown in Eq. (2):

2 $$ Y_{i} \left( {i, R_{s} } \right) = \left\{ {\begin{array}{*{20}l} {R_{s} } & {(R_{s} < 2)} \\ 2 & {\left( {R_{s} \ge 2} \right)} \\ \end{array} } \right., $$

3 $$ Y_{sum} = \mathop \sum \limits_{i = 1}^{11} Yi, $$

where Y_*i*_ represents *i*_th_ peak resolution after normalizing by Eq. (2), and the minimum to maximum response followed by Eq. (3) is 0 to 22, respectively. The randomly experimented fourteen runs to the selected CMAs are tabulated in Table 5 with the studied CMPs levels and designed experimental schedule. To clarify the CCD results, Minitab software ver. 18 was utilized for deriving ANOVA analysis and statistical optimization. Equation (4) is obtained by substituting the experimental data into a mathematical mode encompassing both main effects and interactions reflecting the second-order quadratic polynomial model.

4 $$ Y_{sum} = - \,5.480 + 0.400X_{1} + 2.824X_{4} - 0.0064X_{1} X_{1} - 0.0031X_{1} X_{4} - 0.1037X_{4} X_{4}. $$

**Figure 4:**
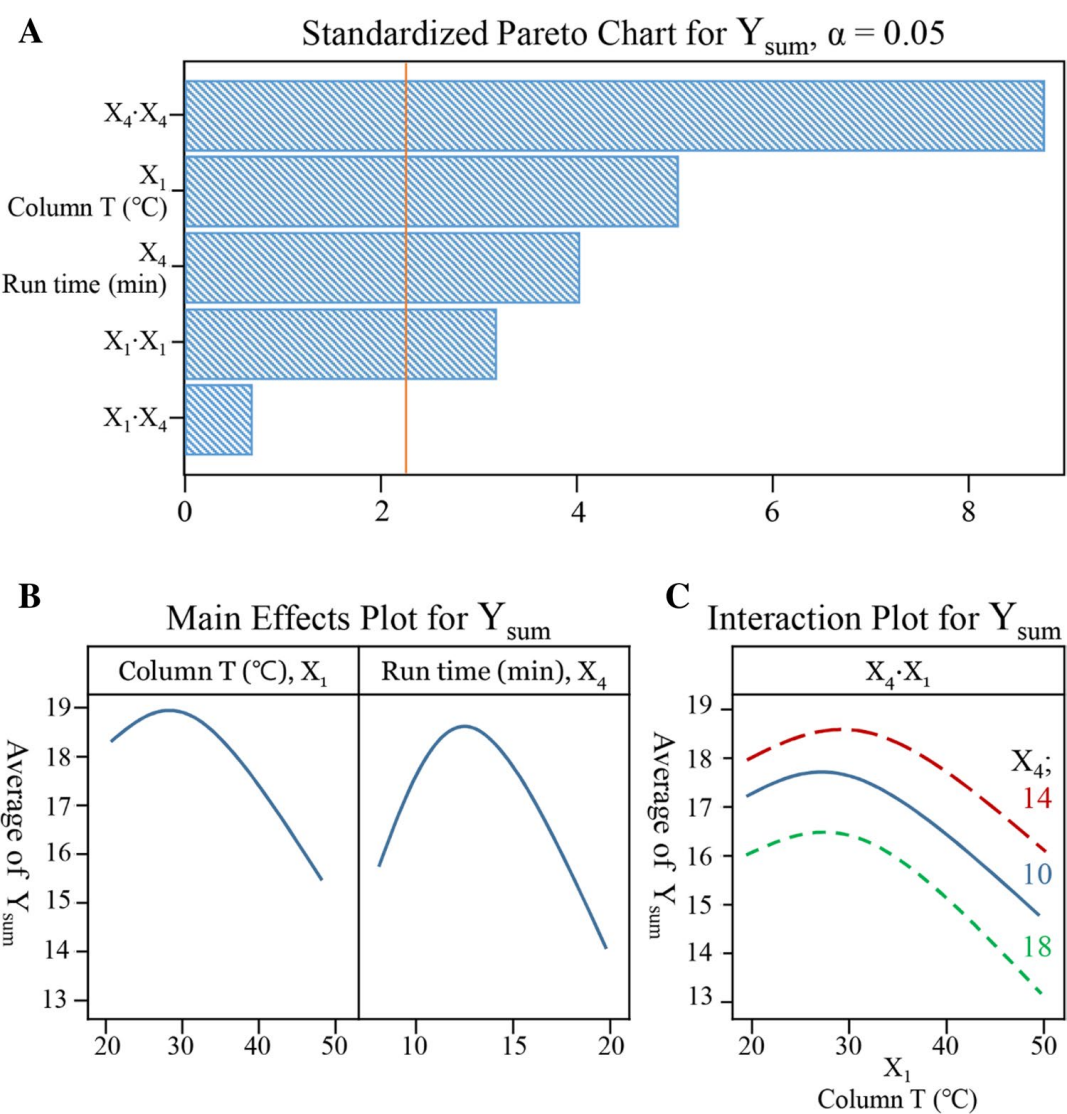
Pareto chart (**A**), main effect plots (**B**), and interaction plots (**C**) obtained during center composite design (CCD) studies of critical method attributes (CMAs), Y_sum_; summarizes the eleven resolutions.

ANOVA analysis was performed to statistically verify the model, which illustrates a statistically highly significant model (*p* < 0.05) and reasonable values of *R*^2^ (95.09% for determination and 90.89% for adjusted). The results are given in Table 4, it is also apparent that two CMPs in the first-order (X_1_, X_4)_ and second-order (X_1_·X_1_, X_4_·X_4)_ terms were significant, whereas the interaction correlation (X_1_·X_4_) was not significant. Those statistical results are also confirmed by observing the Pareto chart, Main effect plots, and Interaction plot shown in Fig. 4.

### Selection of optimum chromatographic solution

**Figure 5:**
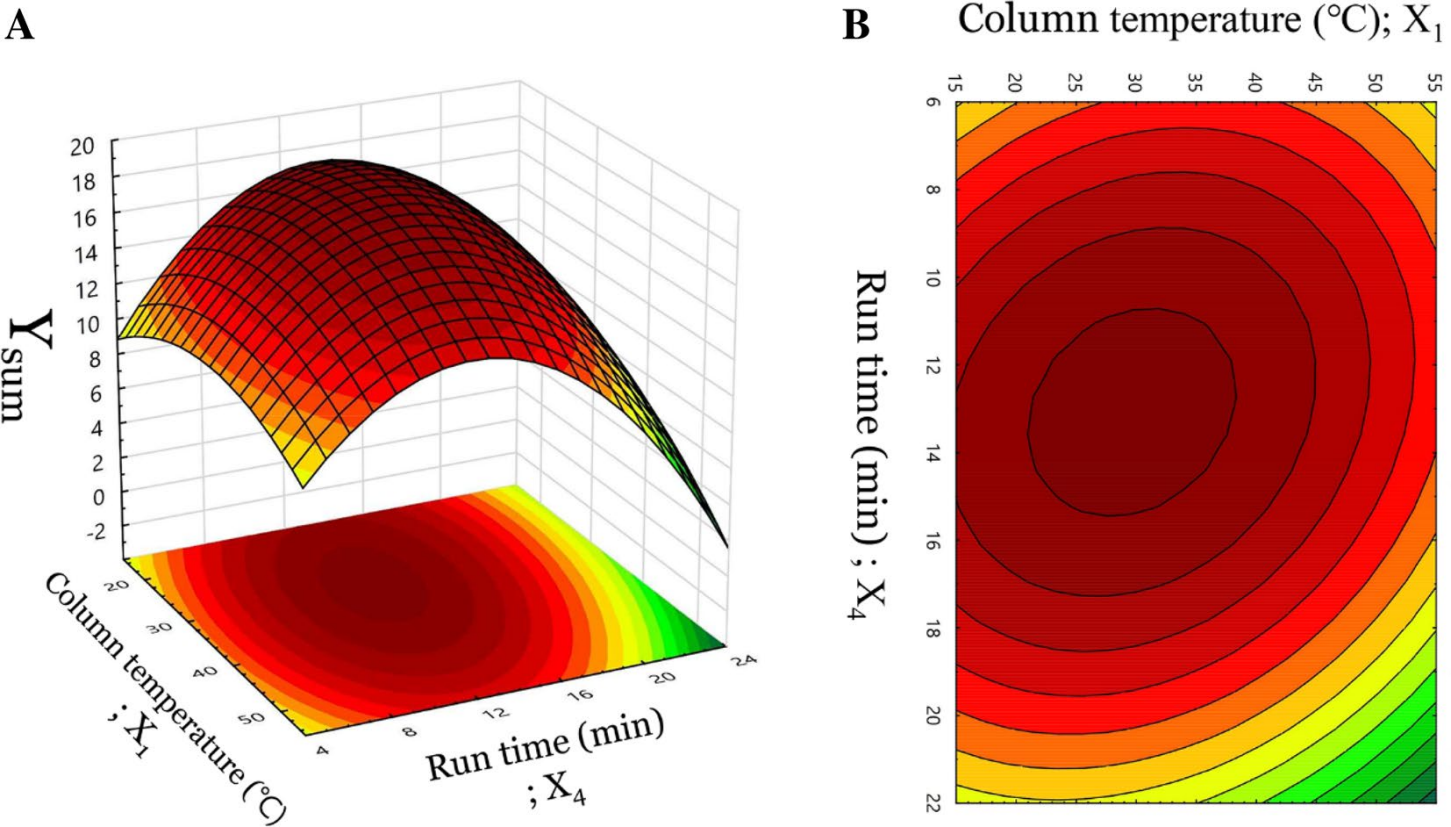
3D response surface plot (**A**) and 2D contour plot (**B**) depicting the interaction of two critical method parameters (CMPs) on the Y_sum_.

**Figure 6:**
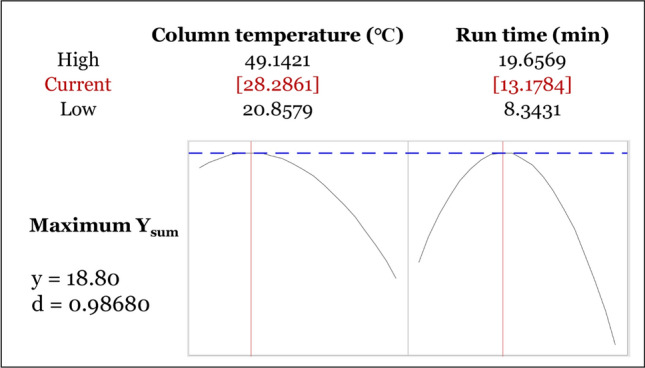
Optimization diagrams calculated mathematically.

To obtain the optimized chromatographic method, the CCD design space was further studied in response surface analysis by using Statistica software ver. 13.3.0, carried out for the specific CMAs, Y_sum_. The 3D response surface (Fig. 5A) and 2D contour plot (Fig. 5B) revealed individual and plausible interaction(s) in factors and responses. Both column temperature (X_1_) and run time (X_4_) have a similarly curved plot, which is gradually increasing and decreasing at around the central level (0). Specifically, the central level of column temperature (X_1_) was 35 ℃ and run time (X_4_) was 14 min, respectively. As observed from Eq. (4), those patterns also may be inferred to be parabolic curves, which mean the response with a maximum value can be calculated by mathematical computing works. Finally, the optimum UHPLC-PDA performance solution with a maximum response Y_sum_ of 18.80 was adjusted mathematically to the column temperature of 28.2861 ℃ and run time of 13.1784 min as portrayed in diagrams in Fig. 6. The verification step was studied to appraise model suitability and the repeatability results were near the predicted value of Y_sum_ with a very acceptable %RSD and %RE (Table 6).

**Table 6:** Predicted and experimental responses at the optimum condition.

Injection number	Predicted Y_sum_	Experimental Y_sum_
1	**18.80**	18.87
2		18.88
3		18.77
4		18.82
5		18.80
6		18.86
	Mean	**18.83**
	SD	0.44
	%RSD	0.23
	%RE	** + 0.16**

*Y*_*sum*_ summarizes the eleven resolutions, *SD* standard deviation, *RSD* relative standard deviation, *RE* relative error.

### Analytical method validation studies

The purpose of validating an analytical method is to demonstrate that the proposed method is suited for its intended use by satisfying the expectations of ATP. At first, method validation of UHPLC fingerprint was performed to determine the precision and stability. The same test solution (30 mg/mL) of the Genkwa Flos, which was injected six times in one day for precision test. Next the same test solution was analyzed 0 and 24 h after the preparation of test solution for stability test. The results were summarized in Supplementary Table S3 as calculated %RSD values of relative retention time (RRT) and relative peak area (RPA) of each peak which were calculated relative to the selected marker peak, apigenin 7-*O*-glucuronide (peak 4). All %RSD values of RRT and RPA of eleven peaks were under 1%, indicating the commendable precision and stability of the fingerprint method.

Next, we studied the quantitative method validation using three standard compounds of apigenin 7-*O*-glucuronide, apigenin, and genkwanin, which were identified as major components by chromatography (Fig. 1). Since the assigned eleven flavonoids were all 2-phenylchromen-4-one backbone flavones, those three peaks with the highest % area in the Fig. 1 were selected as representatives for verification of the optimized analytical method. Standard calibration curves of three compounds for linearity were derived in the range of 0.9765–500.00 μg/mL or 31.25–2000.00 μg/mL with the high values of the coefficient of correlation (0.999), respectively (Table 7). The linear calibration plots with corresponding residual plots are depicted in Supplementary Fig. S1, where none of the points were observed as outliers in the studied range of each concentration. Detection limit (DL) and Quantitation limit (QL) were also drawn out from the linearity test, indicating a sensitive method for quantification of those flavonoids. Precision, a measure of repeatability, was evaluated by intra-day and inter-day variability. As shown in Table 7, the %RSD value of content in the intra-day and also inter-day variability tests were found to be with a reasonable value as under 0.22, respectively. Accuracy of the method was confirmed by spiked and triplicate injections of known standard concentrations into the sample solution. Percentage recovery for the three compounds’ test concentrations studied ranged from 100.13% to 102.49% (Table 7), with their %RSD values less than 0.85.

**Table 7:** Validation results of the method for the determination of apigenin 7-*O*-glucuronide, apigenin, and genkwanin in Genkwa Flos.

Analytes	Regression equation	*R*^2^	Linear range (μg/mL)						
**Calibration curve data in quantitative assay**									
AG	$y = 3783.8x + 48348$	0.999	31.25–2000.00						
A	$y = 7261.6x - 776.69$	0.999	0.9765–500.00						
G	$y = 6741.3x - 4223.1$	0.999	0.9765–500.00						

Analytes	Slope	Standard deviation (*s*_*y/x*_)	DL	QL					
**Detection limit (DL) and quantitation limit (QL)**									
AG	3783.8	25,697.51	22.41	67.92					
A	7261.6	6316.49	2.87	8.70					
G	6741.3	6002.82	2.94	8.90					

Analytes	Intra-day precision (μg/mL, contents; *n* = 6)			Inter-day precision (μg/mL, contents; *n* = 6)					
				Day 1			Day 2		
	AG	A	G	AG	A	G	AG	A	G
**Precision and repeatability test**									
Mean	932.48	129.20	187.89	932.48	129.20	187.97	931.20	129.28	187.97
SD	0.08	0.28	0.29	0.08	0.28	0.37	1.37	0.26	0.37
%RSD	0.01	0.22	0.15	0.01	0.22	0.19	0.15	0.20	0.19

Analytes	Original (μg/mL)	Spiked (μg/mL)	Found (μg/mL)	Recovery (%)	RSD (%)				
**Recovery test in accuracy**									
AG	466.24	125	593.19	100.33	0.19				
		250	728.91	101.77	0.11				
		500	977.85	101.20	0.85				
A	64.60	15.625	80.64	100.51	0.45				
		31.25	95.98	100.14	0.15				
		62.5	127.27	100.13	0.11				
G	93.95	31.25	127.21	101.61	0.39				
		62.5	159.59	102.01	0.11				
		125	224.39	102.49	0.21				

*AG* apigenin 7-*O*-glucuronide, *A* apigenin, *G* Genkwanin, *SD* standard deviation, *RSD* relative Standard deviation, *s*_*y/x*_ the residual standard deviation of the regression line.

## Discussion

System suitability has been checked with the systematically optimized chromatographic method and found to be well within ICH criteria^11^ except resolution, as represented in Fig. 1. Among the eleven flavonoid peaks, resolution of peaks 1, 2, 3, 6, and 9 were under 1.5, which is the remaining challenge for a detailed trial of the isocratic and gradient mixed solvent system or to consider other factors. Meanwhile, an accurate and precise chromatographic method also depends on the %RSD values for injection repeatability precision, tailing factor^9^, plate count^13^, and capacity factor distribution^11^, so those criteria also must be considered as CMAs. However, the only criteria of resolution was selected for CMAs because %RSD and tailing factor were estimated to great precision and symmetry over the entire experiment. Also, when performed CCD studies of those parameters, plate count (> 2000), and capacity factor (> 1), were evaluated as proper in the overall 14 runs of experimental design work as tabulated in Supplementary Table S4.

To apply the AQbD approach, a thorough study on the characteristic of the analyte must be accomplished. The risk assessment studies were conducted carefully to achieve the optimized analytical method that is able to quantify diverse flavonoids from all of the other detected interferences with a substantial acceptable resolution, selectivity, and good efficiency. Thus, optimizing the selected CMPs as column temperature (X_1_) and run time (X_4_) the resolution of eleven identified flavonoid peaks were well resolved as mentioned and represented in Fig. 1.

## Conclusion

The present study adopted a novel AQbD approach to develop a sensitive, robust, and accurate UHPLC-PDA-MS method for the identification and quantification of flavonoids in Genkwa Flos extract. In this approach, a methodical data collection process was conducted to identify the CMPs and CMAs through serial experiments of preliminary tests, risk assessment, full factorial design, and central composite design (CCD). Moreover, a new attempt to express target multiple peak resolutions as a single value was proposed by integrating all analytical peak data, and it provides a direction of how to handle CMAs in developing an analytical method of botanical extracts containing diverse components. The quantitative models depicted by a 3D surface plot with a 2D contour plot between two potential parameters, column temperature (X_1_) and run time (X_4_), were successfully constructed to facilitate finding the most suitable conditions for the chromatographic analysis. In conclusion, an AQbD-based quantitative multi-component analytical method is successfully developed and can serve as a template for other herbal medicinal product cases.

## Material and methods

### Standards and reagents

Apigenin (CAS no. 520-36-5, > 98.6%), apigenin 7-*O*-glucuronide (CAS no. 29741-09-1, > 98.8%), and genkwanin (CAS no. 437-64-9, > 98.0%) were purchased from Chem Faces, Wuhan, China. All of the other reagents were supplied by Duksan Pure Chemicals Co., Ltd., Ilsan, South Korea. For the analytical studies, HPLC-grade water, methanol, and acetonitrile were purchased from Fisher Scientific, Waltham, MA, USA; high purity nitrogen gas was provided by Shinyang Oxygen Co., Ltd., Seoul, South Korea.

### Plant material and preparation of extracts

The flower bud of Daphne genkwa, which is a MFDS (Ministry of Food and Drug Safety of Republic of Korea) certified herbal medicine, was purchased from the Kyung-dong drugstore in Seoul, South Korea. The botanical origin was identified by Prof. Young Pyo Jang who is the head of Medicinal Herb Garden of College of Pharmacy, Kyung Hee University. A Voucher specimen (KHUP-2103) is deposited at the Herbarium of College of Pharmacy, Kyung Hee University, South Korea. Acquiring all plant samples and manufacturing extracts were carried out in compliance with the IUCN Policy Statement on Research Involving Species at Risk of Extinction (https://portals.iucn.org/library/efiles/documents/PP-003-En.pdf) and the Convention on International Trade in Endangered Species of Wild Fauna and Flora https://cites.org. The sample was ground and then powdered with 850 μm mesh sieves. Using 56% acetone in water as the extraction solvent, all flavonoid components were extracted by a shaking extraction procedure. The detailed list of extraction parameters are as follows: agitation speed of 150 rpm, shaking time of 12 h, and extraction temperature of 65 ℃. The concentration of the sample solution was fixed in all experimental sections as 30 mg/mL.

### Instrumentation and UHPLC-PDA-ESI/MS conditions

A Waters AQCUITYTM H-class UPLC system (Waters Corp., Milford, MA, USA) was used for the UHPLC analysis. The system composed of a photo diode array (PDA) detector, quaternary solvent and sample manager, cooling auto sampler, and column oven. The operating software was Empower-3 software (Waters Corp.). A Kinetex-C18 column (2.1 mm × 50 mm i.d., particle size 1.7 μm, Phenomenex, Torrance, CA, USA) was used for all the chromatographic analysis. The sample was maintained at 25 ℃ and the UV/Visible detector wavelength was fixed at 335 nm in all experiments. The mobile phase was composed of acetonitrile and acidified water with 0.1% formic acid. The column oven, flow rate, injection volume, and solvent gradient system were screened by experimental design.

To identify and assign flavonoids, the mass spectrometric studies were carried out on an AccuTOF^®^ single-reflection TOF mass spectrometer (JEOL, Tokyo, Japan) equipped with an ESI probe. Some important parameters of mass spectrometry were as follows: positive ion mode, mass range m/z 100—1500, needle voltage—2000 V, orifice-1 voltage—80 V, ring lens voltage—10 V, orifice-2 voltage—5 V. Nebulizing and desolvation gas was nitrogen. The desolvation temperature was 250 °C and the orifice-1 temperature was set to 80 °C. Mass Center System (version 1.3.7b, JEOL, Tokyo, Japan) was operating software and mass calibration was conducted using the YOKUDELNA kit (JEOL, Tokyo, Japan).

### Statistical analysis

In current study, two design of experiments, full factorial design (FFD) and central composite design (CCD), were constructed and also statistical analyzed using Minitab software ver. 18 (Minitab Inc., State College, PA, USA). The statistically significant coefficients (*p* < 0.05) per analysis of variance (ANOVA) were used in framing the polynomial equation followed by the evaluation of the fit of the two models. Parameters evaluated for appropriate fitting of the models including coefficient of correlation (R^2^), lack of fit, F-value, and P-value are listed, respectively. Among them, the result of CCD was also studied in response surface analysis utilizing Statistica software ver. 13.3.0 (TIBCO Software Inc., Palo Alto, CA, USA).

### Chromatographic method validation analysis

After defining the design model, the analytical operating point was validated per the International Conference on Harmonization (ICH) guideline Q2 (R1) and the parameters are described below^33^. Among the eleven identified flavonoids, three major eluates were chosen for study in this validation process, which are apigenin 7-*O*-glucuronide, apigenin, and genkwanin.

### Linearity and range

To confirm linearity, working standards of apigenin 7-*O*-glucuronide in the range of 31.25–2000.00 μg/mL, apigenin and genkwanin in the range of 0.9765–500.00 μg/mL were prepared by a serial dilution process and then analyzed. From regression analysis, three regression lines along with the regression equation and least squares were derived by each of the standard compounds, respectively.

### Detection limit and quantitation limit

Following the guideline Q2 (R1), there are several approaches for calculating Detection limit (DL) and Quantitation limit (QL), we chose the method “Based on the Standard Deviation of the Response (*s*) and the Slope (α)^33^” for this study. In Eqs. (5) and (6), the slope (*α*) was derived from each slope of the three analytical curves. The standard deviation of the response (*s*) was determined based on the residual standard deviation of each regression line.

5 $$ {\text{DL}} = 3.3 \times \frac{s}{\alpha}, $$

6 $$ {\text{QL}} = 10 \times \frac{s}{\alpha}. $$

### Precision

Repeatability and Intermediate Precision were performed with a known concentration of the analyte (30 mg/mL) to investigate precision. On the same day, two samples at 100% of the test concentration were studied by six determinations each for the repeatability test. One sample was prepared for chromatographic analysis by six determinations on the next day testing for Intermediate Precision. All results were assessed as the percentage relative error by converted reference contents.

### Accuracy

Calculating the percentage recovery of analyzed spiked samples was used for the accuracy test. Three known amount of each standard solutions: 125, 250, and 500 μg/mL of apigenin 7-*O*-glucuronide; 15.625, 31.25, and 62.5 μg/mL of apigenin; 31.25, 62.5, and 125 μg/mL of genkwanin were spiked with respect to the analyte (30 mg/mL) solution. The recovery studies were carried out three times showing that the percentage recovery and also percentage relative error were calculated to be accurate.

## Supplementary Information

Supplementary Information.
