# Supplementary material for: Analytical quality by design methodology for botanical raw material analysis: a case study of flavonoids in Genkwa Flos
Source: Sci Rep. 2021 Jun 7;11:11936. doi: 10.1038/s41598-021-91341-w (PMC8185112; doi:10.1038/s41598-021-91341-w)
Supplement: Supplementary file 1 — Supplementary Information. [file 41598_2021_91341_MOESM1_ESM.docx]

**Supplementary Information**

**Analytical quality by design methodology for botanical raw material analysis: A case study of flavonoids in Genkwa Flos**

Min Kyoung Kim^1^, Sang Cheol Park^2^, Geonha Park^2^, Eunjung Choi^2^, Yura Ji^2^, Young Pyo Jang^2,3,*^

^1^University Institute of Pharmacy, Kyung Hee University, Seoul 02447, South Korea. ^2^Department of Life and Nanopharmaceutical Sciences, Graduate school, Kyung Hee University, Seoul 02447, South Korea.

^3^Department of Oriental Pharmaceutical Science, College of Pharmacy, Kyung Hee University, Seoul 02447, South Korea.

**^*^**Corresponding author

Email: [ypjang@khu.ac.kr](mailto:ypjang@khu.ac.kr)

Address: Department of Oriental Pharmaceutical Science, College of Pharmacy, Kyung Hee University, Seoul 02447, South Korea

Tel: 82-2-961-9421

Fax: 82-2-961-9580

**Table S1:** Analytical Target Profile (ATP) for UHPLC method development of Genkwa Flos

| **ATP elements** | **Objective(s)** | **Explanation** |
| --- | --- | --- |
| Target sample | Genkwa Flos | Analytical method development for the quantitatively detect of flavonoids in Genkwa Flos |
| Analytical technique | RP-UHPLC | UHPLC technique utilized with reversed-phase non-polar stationary phase providing improved retention for the majority of diverse phytochemicals like flavonoids |
| Instrument requirement | Auto-sampler | Auto-sampler provides accurate injection |
|  | Quaternary pump system | Quaternary pump system for precise mixing of mobile phase |
|  | PDA detector | PDA detector scans spectrum each analyte and also measurements at all wavelengths |
| Sample preparation | Liquid state | Analyte should be prepared in liquid state for ensuring absolute miscibility with mobile phase |
| Target method application | Estimate of flavonoids | The developed method is applicable to detect flavonoids in Genkwa Flos in a reasonably well separated result |

**Table S2:** Preliminary screening results

| **Parameters** | **Experimental strategy** | **Inference drawn** |
| --- | --- | --- |
| Different columns | ACQUITY UPLC^®^ BEH C_18_ column  (2.1 × 50 mm i.d., 1.7 μm) | There were no special differences when comparing those four different columns, but finally, one was selected in consideration of the peak shape, %RSD, plate count, capacity factor, and run time. |
|  | Endeavorsil^TM^ C18 column  (2.1 × 50 mm i.d., 1.8 μm) |  |
|  | Kinetex C_18_ column  (2.1 × 100 mm i.d., 1.7 μm) |  |
|  | ***Kinetex C_18_ column**  **(2.1 × 50 mm i.d., 1.7 μm)** |  |
| Various combination of solvents | Methanol and non-acidified water | An uneven baseline of chromatogram in gradient elution |
|  | Acetonitrile and non-acidified water | Poor peak symmetry; particular in tailing |
|  | Acetonitrile and 0.1% acetic acid | Poor resolution of substances; Some peaks were not fully separated from a neighboring |
|  | ***Acetonitrile and 0.1% formic acid** | Improved peak shape and resolution |
| Detection wavelength | 210 nm | An uneven baseline of chromatogram in gradient elution |
|  | 254 nm | Decrease specificity of substances due to the detection of high polar peaks |
|  | ***335 nm** | Provide maximal specific detection to flavonoids |
| *Selected conditions of each parameter | | |

**Table S3:** Relative retention time and relative peak area of 11 peaks compare to marker peak of apigenin 7-*O*-glucuronide.

| ***RRTs obtained from precision and stability test** | | | | | |
| --- | --- | --- | --- | --- | --- |
| Peak no. | RRT (*n* = 6) | Stability (*n* = 6) | | Average | %RSD |
|  |  | 0 hour | 24 hours |  |  |
| **1** | 0.771 | 0.771 | 0.771 | 0.771 | 0.011 |
| **2** | 0.785 | 0.785 | 0.785 | 0.785 | 0.012 |
| **3** | 0.846 | 0.846 | 0.846 | 0.846 | 0.005 |
| *Marker peak;* **4**  apigenin 7-*O*-glucuronide | **1.000** | **1.000** | **1.000** | **1.000** | **0.000** |
| **5** | 1.075 | 1.075 | 1.075 | 1.075 | 0.013 |
| **6** | 1.163 | 1.163 | 1.163 | 1.163 | 0.015 |
| **7** | 1.404 | 1.404 | 1.404 | 1.404 | 0.016 |
| **8** | 1.565 | 1.565 | 1.566 | 1.565 | 0.016 |
| **9** | 1.705 | 1.705 | 1.706 | 1.705 | 0.015 |
| **10** | 2.106 | 2.107 | 2.107 | 2.107 | 0.035 |
| **11** | 2.481 | 2.482 | 2.482 | 2.482 | 0.038 |
| ***RPAs obtained from precision and stability test** | | | | | |
| Peak no. | RPA (*n* = 6) | Stability (*n* = 6) | | Average | %RSD |
|  |  | 0 hour | 24 hours |  |  |
| **1** | 0.035 | 0.035 | 0.035 | 0.035 | 0.356 |
| **2** | 0.046 | 0.046 | 0.045 | 0.046 | 0.202 |
| **3** | 0.102 | 0.102 | 0.102 | 0.102 | 0.165 |
| *Marker peak;* **4**  apigenin 7-*O*-glucuronide | **1.000** | **1.000** | **1.000** | **1.000** | **0.000** |
| **5** | 0.198 | 0.198 | 0.198 | 0.198 | 0.118 |
| **6** | 0.075 | 0.076 | 0.074 | 0.075 | 0.947 |
| **7** | 0.072 | 0.073 | 0.072 | 0.073 | 0.138 |
| **8** | 0.138 | 0.138 | 0.138 | 0.138 | 0.064 |
| **9** | 0.815 | 0.815 | 0.813 | 0.814 | 0.167 |
| **10** | 0.226 | 0.226 | 0.226 | 0.226 | 0.070 |
| **11** | 0.504 | 0.504 | 0.503 | 0.504 | 0.144 |
| *RRT; relative retention time  *RPA; relative peak area | | | | | |

**Table S4:** The calculated USP Plate Count and USP Capacity factor in every fourteen experimental runs by central composite design (CCD) matrix

| **14 runs by CCD**  **Chromatographic criteria** | | **Peak no.** | | | | | | | | | | |
| --- | --- | --- | --- | --- | --- | --- | --- | --- | --- | --- | --- | --- |
|  |  | **1** | **2** | **3** | **4** | **5** | **6** | **7** | **8** | **9** | **10** | **11** |
| **1** | USP Plate Count | 50509 | 53401 | 60151 | 83109 | 89555 | 103831 | 159726 | 188207 | 230619 | 336201 | 463022 |
|  | USP Capacity factor | 11.65 | 12.00 | 12.80 | 15.22 | 15.84 | 17.13 | 21.49 | 23.41 | 26.02 | 31.63 | 37.29 |
| **2** | USP Plate Count | 40550 | 41990 | 48778 | 68196 | 78881 | 92242 | 134542 | 167211 | 198458 | 302940 | 420348 |
|  | USP Capacity factor | 10.33 | 10.53 | 11.43 | 13.69 | 14.80 | 16.09 | 19.64 | 22.01 | 24.07 | 29.97 | 35.48 |
| **3** | USP Plate Count | 40596 | 42084 | 48854 | 68315 | 79009 | 92381 | 134710 | 167398 | 198712 | 303129 | 420645 |
|  | USP Capacity factor | 10.34 | 10.54 | 11.44 | 13.71 | 14.82 | 16.10 | 19.65 | 22.02 | 24.08 | 29.98 | 35.50 |
| **4** | USP Plate Count | 40573 | 42060 | 48854 | 68315 | 78977 | 92347 | 134710 | 167398 | 198661 | 303066 | 420645 |
|  | USP Capacity factor | 10.33 | 10.54 | 11.44 | 13.71 | 14.81 | 16.10 | 19.65 | 22.02 | 24.08 | 29.98 | 35.50 |
| **5** | USP Plate Count | 27461 | 33667 | 33667 | 48024 | 63159 | 74763 | 99423 | 135592 | 152769 | 252578 | 356102 |
|  | USP Capacity factor | 8.48 | 9.49 | 9.49 | 11.53 | 13.37 | 14.64 | 17.03 | 20.06 | 21.35 | 27.74 | 33.13 |
| **6** | USP Plate Count | 34024 | 52349 | 63073 | 91515 | 112244 | 131417 | 193047 | 257194 | 295315 | 470910 | 667256 |
|  | USP Capacity factor | 9.41 | 11.92 | 13.18 | 16.08 | 17.91 | 19.46 | 23.80 | 27.63 | 29.68 | 37.74 | 45.11 |
| **7** | USP Plate Count | 27841 | 29427 | 32813 | 43502 | 46878 | 54462 | 78080 | 88804 | 109315 | 156952 | 211180 |
|  | USP Capacity factor | 8.45 | 8.72 | 9.26 | 10.81 | 11.26 | 12.22 | 14.83 | 15.88 | 17.72 | 21.44 | 25.03 |
| **8** | USP Plate Count | 40573 | 41966 | 48753 | 68196 | 78881 | 92242 | 134542 | 167117 | 198356 | 302814 | 420274 |
|  | USP Capacity factor | 10.33 | 10.53 | 11.42 | 13.69 | 14.80 | 16.09 | 19.64 | 22.00 | 24.06 | 29.96 | 35.48 |
| **9** | USP Plate Count | 22931 | 35970 | 44461 | 64894 | 86470 | 102217 | 139876 | 196680 | 218810 | 367097 | 524259 |
|  | USP Capacity factor | 7.69 | 9.88 | 11.10 | 13.62 | 15.87 | 17.34 | 20.46 | 24.45 | 25.84 | 33.76 | 40.54 |
| **10** | USP Plate Count | 25417 | 26078 | 30475 | 41616 | 49411 | 57957 | 78913 | 97487 | 114940 | 176592 | 242064 |
|  | USP Capacity factor | 8.12 | 8.24 | 8.98 | 10.67 | 11.71 | 12.77 | 15.07 | 16.86 | 18.39 | 23.03 | 27.14 |
| **11** | USP Plate Count | 40550 | 41990 | 48778 | 68225 | 78913 | 92277 | 134584 | 167211 | 198407 | 302940 | 420497 |
|  | USP Capacity factor | 10.33 | 10.53 | 11.43 | 13.70 | 14.81 | 16.09 | 19.64 | 22.01 | 24.06 | 29.97 | 35.49 |
| **12** | USP Plate Count | 38595 | 41313 | 45406 | 60657 | 62786 | 72623 | 110300 | 123181 | 154337 | 217049 | 293516 |
|  | USP Capacity factor | 10.05 | 10.44 | 10.99 | 12.86 | 13.10 | 14.16 | 17.69 | 18.75 | 21.11 | 25.22 | 29.49 |
| **13** | USP Plate Count | 40596 | 42037 | 48803 | 68225 | 78881 | 92277 | 134584 | 167211 | 198458 | 302940 | 420423 |
|  | USP Capacity factor | 10.34 | 10.54 | 11.43 | 13.70 | 14.80 | 16.09 | 19.64 | 22.01 | 24.07 | 29.97 | 35.49 |
| **14** | USP Plate Count | 62729 | 65595 | 75295 | 106574 | 118611 | 137620 | 213550 | 262554 | 315138 | 471537 | 657582 |
|  | USP Capacity factor | 13.14 | 13.46 | 14.49 | 17.43 | 18.44 | 19.94 | 25.09 | 27.93 | 30.69 | 37.76 | 44.78 |

**
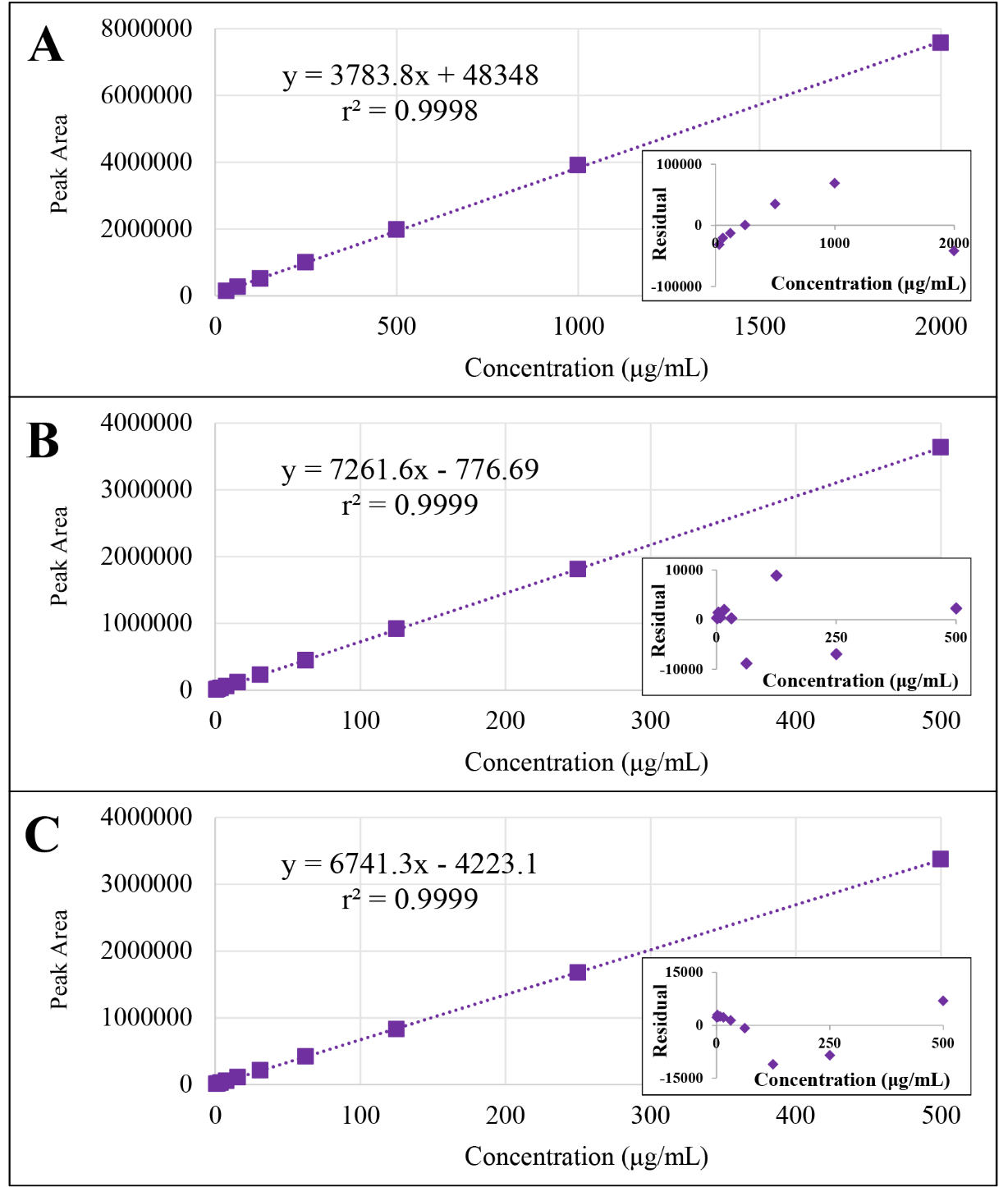
**

**Figure S1.** Linear calibration plots for liquid chromatographic estimation of apigenin 7-*O*-glucuronide (A), apigenin (B), and genkwanin. The corresponding insets depict the residual plots confirming the linearity range.
